# {1,3-Bis[(diphenyl­phosphanyl-κ*P*)­oxy]prop-2-yl-κ*C* ^2^}iodido(trimethyl­phosphane)cobalt(II)

**DOI:** 10.1107/S1600536810013747

**Published:** 2010-04-21

**Authors:** Guoqiang Xu, Xiaoyan Li

**Affiliations:** a School of Chemistry and Chemical Engineering, Shandong University, Jinan 250100, People’s Republic of China

## Abstract

The title compound, [Co(C_27_H_25_O_2_P_2_)I(C_3_H_9_P)], was synthesized by the addition of 1-iodo­butane to a solution of the parent cobalt complex {1,3-bis­[(diphenyl­phosphan­yl)­oxy]prop-2-yl}bis­(trimethyl­phosphane)cobalt(II). Two five-membered cobaltocycles with considerable ring bending (sum of inter­nal angles = 516.4 and 517.7°) are formed through two P atoms of the PPh_2_ groups and a metallated C*sp* ^3^ atom. The Co^II^ atom is centered in a trigonal-bipyramidal configuration.

## Related literature

For general background to transition metal complexes with PCP pincer ligands and their preparation, see: Boom & Milstein (2003 ▶); Pandarus *et al.* (2008 ▶); Xu *et al.* (2009 ▶); Zheng *et al.* (2009 ▶). For Co—C*sp* ^3^ bond lengths, see: Klein *et al.* (2003 ▶).
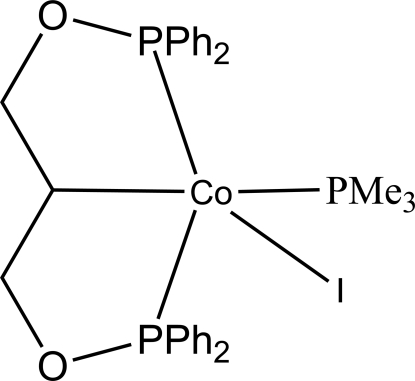


## Experimental

### 

#### Crystal data

[Co(C_27_H_25_O_2_P_2_)I(C_3_H_9_P)]
*M* *_r_* = 705.31
Orthorhombic, 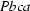

*a* = 15.161 (3) Å
*b* = 18.194 (4) Å
*c* = 21.410 (4) Å
*V* = 5906 (2) Å^3^
*Z* = 8
Mo *K*α radiation
μ = 1.82 mm^−1^
*T* = 293 K
0.20 × 0.15 × 0.10 mm

#### Data collection

Bruker SMART CCD area-detector diffractometer
Absorption correction: multi-scan (*SADABS*; Sheldrick, 2004 ▶) *T*_min_ = 0.713, *T*_max_ = 0.876
35496 measured reflections
6237 independent reflections
5551 reflections with *I* > 2σ(*I*)
*R*_int_ = 0.071

#### Refinement

*R*[*F* ^2^ > 2σ(*F* ^2^)] = 0.024
*wR*(*F* ^2^) = 0.061
*S* = 1.04
6237 reflections
357 parameters
H atoms treated by a mixture of independent and constrained refinement
Δρ_max_ = 0.57 e Å^−3^
Δρ_min_ = −0.54 e Å^−3^

### 

Data collection: *SMART* (Bruker, 1997 ▶); cell refinement: *SAINT* (Bruker, 1997 ▶); data reduction: *SAINT*; program(s) used to solve structure: *SHELXS97* (Sheldrick, 2008 ▶); program(s) used to refine structure: *SHELXL97* (Sheldrick, 2008 ▶); molecular graphics: *SHELXTL* (Sheldrick, 2008 ▶); software used to prepare material for publication: *SHELXTL*.

## Supplementary Material

Crystal structure: contains datablocks I, global. DOI: 10.1107/S1600536810013747/zq2034sup1.cif

Structure factors: contains datablocks I. DOI: 10.1107/S1600536810013747/zq2034Isup2.hkl

Additional supplementary materials:  crystallographic information; 3D view; checkCIF report

## Figures and Tables

**Fig. 1.**
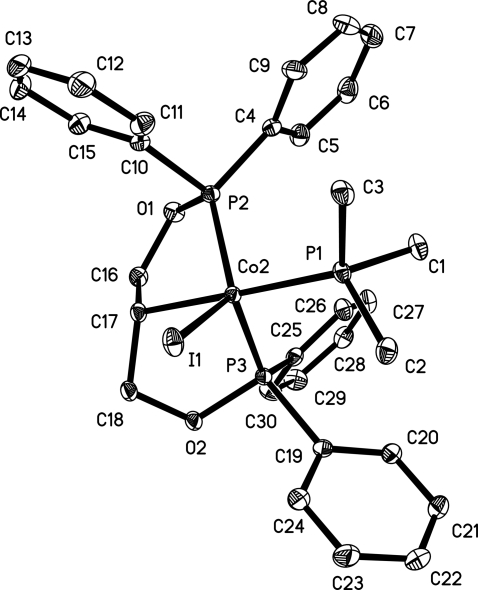
The molecular structure of the title molecule showing the atom-labelling scheme. Thermal ellipsoids are drawn at the 30% probability level. All hydrogen atoms are omitted for clarity.

## supplementary crystallographic information

### Comment

Transition metal complexes with PCP pincer ligands have attracted a substantial amount of interest (Boom *et al.* 2003). We previously reported that the central sp^3^ C—H bond of (Ph_2_POCH_2_)_2_CH_2_ could be activated by Co(PMe_3_)_4_Me to afford metallated PCP pincer compounds at room temperature (Xu *et al.*, 2009) and the subsequent reaction with CH_3_I gave rise to iodomethylcobalt(III) complex. Here we explored the reaction of Co(C_27_H_25_O_2_P_2_)(C_3_H_9_P)_2_ with n-C_4_H_9_I, which afforded the title compound via one-electron oxidative addition. The rest part of products might be C,*C*-coupling product (Zheng *et al.*, 2009), despite it has not been isolated.

The molecular structure is shown in Fig. 1. The Co^II^ atom is five coordinated in a trigonal bipyramidal configuration. The Co—C bond distance of 2.068 (18) Å is within the range of Co—C (sp^3^) bonds (2.03-2.15 Å) (Klein *et al.*, 2003).

### Experimental

Standard vacuum techniques were used in manipulations of volatile and air sensitive material. The title compound was synthesized by combining a solution of {1,3-bis[(diphenylphosphanyl)oxy]prop-2-yl}bis(trimethylphosphane)cobalt(II) (733 mg, 1.12 mmol) in 30 ml of diethyl ether with a sample of n-C_4_H_9_I (203 mg, 1.12 mmol) in 30 ml of diethyl ether at 273 K. After kept stirring for 16 h at room temperature, the color changed from red to brown. Volatiles were concentrated and filtrated. Red crystals, which were suitable for X-ray diffraction, could be obtained from diethyl ether at 255 K.

### Refinement

The H atoms bound to C16-C18 were located in a difference Fourier map and refined isotropically. The remaining H atoms were included in calculated positions, with C—H = 0.93 Å (aromatic) and 0.96 Å (alkyl), and with *U*_iso_(H) = 1.2 (1.5 for alkyl groups) times *U*_eq_(C).

### Crystal data

**Table:**

[Co(C_27_H_25_O_2_P_2_)I(C_3_H_9_P)]	*F*(000) = 2840
*M**_r_* = 705.31	*D*_x_ = 1.587 Mg m^−^^3^
Orthorhombic, *P**b**c**a*	Mo *K*α radiation, λ = 0.71073 Å
Hall symbol: -P 2ac 2ab	Cell parameters from 13063 reflections
*a* = 15.161 (3) Å	θ = 2.0–26.9°
*b* = 18.194 (4) Å	µ = 1.82 mm^−^^1^
*c* = 21.410 (4) Å	*T* = 293 K
*V* = 5906 (2) Å^3^	Block, red
*Z* = 8	0.20 × 0.15 × 0.10 mm

### Data collection

**Table:**

Bruker SMART CCD area-detector diffractometer	6237 independent reflections
Radiation source: fine-focus sealed tube	5551 reflections with *I* > 2σ(*I*)
graphite	*R*_int_ = 0.071
phi and ω scans	θ_max_ = 26.7°, θ_min_ = 2.0°
Absorption correction: multi-scan (*SADABS*; Sheldrick, 2004)	*h* = −19→18
*T*_min_ = 0.713, *T*_max_ = 0.876	*k* = −23→23
35496 measured reflections	*l* = −27→20

### Refinement

**Table:**

Refinement on *F*^2^	Primary atom site location: structure-invariant direct methods
Least-squares matrix: full	Secondary atom site location: difference Fourier map
*R*[*F*^2^ > 2σ(*F*^2^)] = 0.024	Hydrogen site location: inferred from neighbouring sites
*wR*(*F*^2^) = 0.061	H atoms treated by a mixture of independent and constrained refinement
*S* = 1.04	*w* = 1/[σ^2^(*F*_o_^2^) + (0.0293*P*)^2^ + 1.1856*P*] where *P* = (*F*_o_^2^ + 2*F*_c_^2^)/3
6237 reflections	(Δ/σ)_max_ = 0.004
357 parameters	Δρ_max_ = 0.57 e Å^−^^3^
0 restraints	Δρ_min_ = −0.54 e Å^−^^3^

### Special details

**Table:**

Geometry. All esds (except the esd in the dihedral angle between two l.s. planes) are estimated using the full covariance matrix. The cell esds are taken into account individually in the estimation of esds in distances, angles and torsion angles; correlations between esds in cell parameters are only used when they are defined by crystal symmetry. An approximate (isotropic) treatment of cell esds is used for estimating esds involving l.s. planes.
Refinement. Refinement of *F*^2^ against ALL reflections. The weighted *R*-factor wR and goodness of fit *S* are based on *F*^2^, conventional *R*-factors *R* are based on *F*, with *F* set to zero for negative *F*^2^. The threshold expression of *F*^2^ > σ(*F*^2^) is used only for calculating *R*-factors(gt) etc. and is not relevant to the choice of reflections for refinement. *R*-factors based on *F*^2^ are statistically about twice as large as those based on *F*, and *R*- factors based on ALL data will be even larger.

### Fractional atomic coordinates and isotropic or equivalent isotropic displacement parameters (Å^2^)

**Table:**

	*x*	*y*	*z*	*U*_iso_*/*U*_eq_	
I1	0.657194 (9)	0.086157 (8)	0.506996 (6)	0.02926 (5)	
Co2	0.644969 (16)	0.062554 (12)	0.387635 (11)	0.01625 (6)	
P1	0.75443 (3)	0.14172 (2)	0.37052 (2)	0.02092 (10)	
P2	0.54174 (3)	0.10487 (2)	0.32815 (2)	0.01924 (10)	
P3	0.70421 (3)	−0.03885 (2)	0.35418 (2)	0.01858 (10)	
O1	0.48603 (9)	0.03615 (7)	0.30021 (7)	0.0249 (3)	
O2	0.65551 (9)	−0.10523 (7)	0.39172 (7)	0.0247 (3)	
C1	0.79921 (15)	0.15169 (11)	0.29188 (10)	0.0300 (4)	
H1A	0.8427	0.1900	0.2916	0.045*	
H1B	0.7525	0.1640	0.2635	0.045*	
H1C	0.8260	0.1063	0.2792	0.045*	
C2	0.85631 (13)	0.12727 (12)	0.41438 (11)	0.0302 (4)	
H2A	0.8833	0.0821	0.4012	0.045*	
H2B	0.8431	0.1247	0.4582	0.045*	
H2C	0.8960	0.1674	0.4068	0.045*	
C3	0.72818 (15)	0.23686 (10)	0.39039 (10)	0.0291 (4)	
H3A	0.7813	0.2655	0.3906	0.044*	
H3B	0.7014	0.2385	0.4310	0.044*	
H3C	0.6880	0.2565	0.3600	0.044*	
C4	0.55196 (12)	0.16068 (10)	0.25779 (9)	0.0235 (4)	
C5	0.56173 (14)	0.12717 (12)	0.20005 (10)	0.0297 (4)	
H5	0.5582	0.0763	0.1968	0.036*	
C6	0.57680 (15)	0.16956 (14)	0.14697 (11)	0.0380 (5)	
H6	0.5825	0.1469	0.1083	0.046*	
C7	0.58336 (16)	0.24521 (14)	0.15141 (13)	0.0437 (6)	
H7	0.5942	0.2733	0.1160	0.052*	
C8	0.57375 (18)	0.27844 (13)	0.20852 (13)	0.0424 (6)	
H8	0.5788	0.3292	0.2117	0.051*	
C9	0.55662 (16)	0.23727 (11)	0.26161 (11)	0.0330 (5)	
H9	0.5482	0.2606	0.2998	0.040*	
C10	0.45903 (13)	0.15345 (10)	0.37462 (9)	0.0240 (4)	
C11	0.48287 (15)	0.20811 (11)	0.41667 (11)	0.0327 (5)	
H11	0.5416	0.2224	0.4196	0.039*	
C12	0.42052 (18)	0.24143 (12)	0.45408 (12)	0.0417 (6)	
H12	0.4372	0.2786	0.4815	0.050*	
C13	0.33319 (18)	0.21962 (14)	0.45081 (13)	0.0458 (7)	
H13	0.2910	0.2420	0.4760	0.055*	
C14	0.30906 (17)	0.16476 (15)	0.41022 (14)	0.0449 (6)	
H14	0.2505	0.1497	0.4084	0.054*	
C15	0.37125 (15)	0.13148 (12)	0.37182 (11)	0.0332 (5)	
H15	0.3542	0.0946	0.3443	0.040*	
C16	0.49139 (13)	−0.02708 (10)	0.34158 (10)	0.0236 (4)	
C17	0.54239 (12)	−0.01073 (9)	0.40104 (9)	0.0207 (4)	
C18	0.57921 (13)	−0.08121 (10)	0.42799 (10)	0.0243 (4)	
C19	0.81877 (13)	−0.05789 (9)	0.37445 (10)	0.0229 (4)	
C20	0.88739 (14)	−0.05669 (10)	0.33109 (10)	0.0261 (4)	
H20	0.8749	−0.0501	0.2889	0.031*	
C21	0.97401 (14)	−0.06518 (12)	0.35030 (12)	0.0337 (5)	
H21	1.0193	−0.0644	0.3211	0.040*	
C22	0.99311 (16)	−0.07482 (13)	0.41270 (13)	0.0400 (5)	
H22	1.0513	−0.0806	0.4254	0.048*	
C23	0.92562 (17)	−0.07593 (14)	0.45670 (12)	0.0428 (6)	
H23	0.9387	−0.0820	0.4988	0.051*	
C24	0.83859 (15)	−0.06791 (12)	0.43761 (12)	0.0333 (5)	
H24	0.7934	−0.0692	0.4670	0.040*	
C25	0.69869 (13)	−0.07007 (10)	0.27348 (9)	0.0216 (4)	
C26	0.71675 (14)	−0.02226 (10)	0.22455 (10)	0.0272 (4)	
H26	0.7265	0.0273	0.2328	0.033*	
C27	0.72045 (14)	−0.04749 (12)	0.16343 (10)	0.0313 (4)	
H27	0.7343	−0.0153	0.1312	0.038*	
C28	0.70351 (14)	−0.12086 (12)	0.15053 (10)	0.0299 (4)	
H28	0.7070	−0.1382	0.1097	0.036*	
C29	0.68137 (16)	−0.16818 (11)	0.19879 (11)	0.0336 (5)	
H29	0.6677	−0.2169	0.1901	0.040*	
C30	0.67953 (16)	−0.14335 (10)	0.25967 (11)	0.0302 (4)	
H30	0.6654	−0.1757	0.2918	0.036*	
H18	0.5970 (16)	−0.0751 (11)	0.4712 (12)	0.021 (5)*	
H31	0.5038 (15)	0.0134 (12)	0.4328 (11)	0.023 (5)*	
H16	0.5197 (16)	−0.0676 (12)	0.3174 (11)	0.025 (6)*	
H17	0.4303 (17)	−0.0420 (13)	0.3517 (12)	0.029 (6)*	
H19	0.5380 (17)	−0.1227 (13)	0.4249 (12)	0.032 (6)*	

### Atomic displacement parameters (Å^2^)

**Table:**

	*U*^11^	*U*^22^	*U*^33^	*U*^12^	*U*^13^	*U*^23^
I1	0.03046 (8)	0.03908 (9)	0.01822 (7)	−0.00978 (5)	0.00190 (5)	−0.00184 (5)
Co2	0.01717 (12)	0.01357 (11)	0.01801 (12)	−0.00099 (8)	0.00126 (9)	−0.00041 (8)
P1	0.0226 (2)	0.0175 (2)	0.0227 (2)	−0.00525 (17)	0.00225 (19)	−0.00124 (17)
P2	0.0203 (2)	0.01627 (19)	0.0211 (2)	0.00030 (16)	−0.00108 (18)	−0.00069 (16)
P3	0.0197 (2)	0.01471 (19)	0.0214 (2)	0.00055 (16)	0.00333 (18)	0.00018 (16)
O1	0.0270 (7)	0.0225 (6)	0.0252 (7)	−0.0043 (5)	−0.0052 (6)	−0.0015 (5)
O2	0.0255 (7)	0.0170 (6)	0.0317 (8)	0.0013 (5)	0.0089 (6)	0.0035 (5)
C1	0.0343 (12)	0.0263 (9)	0.0293 (11)	−0.0095 (8)	0.0085 (9)	−0.0016 (8)
C2	0.0243 (10)	0.0310 (10)	0.0353 (12)	−0.0068 (8)	0.0002 (9)	−0.0028 (9)
C3	0.0342 (11)	0.0187 (8)	0.0343 (11)	−0.0064 (8)	0.0039 (9)	−0.0033 (8)
C4	0.0192 (9)	0.0259 (9)	0.0254 (10)	0.0026 (7)	−0.0018 (8)	0.0041 (7)
C5	0.0265 (10)	0.0345 (10)	0.0281 (11)	−0.0025 (8)	−0.0002 (8)	−0.0009 (8)
C6	0.0299 (12)	0.0571 (14)	0.0270 (11)	−0.0026 (10)	0.0011 (9)	0.0020 (10)
C7	0.0361 (13)	0.0555 (14)	0.0394 (13)	0.0018 (11)	0.0003 (10)	0.0241 (11)
C8	0.0496 (15)	0.0306 (10)	0.0470 (15)	0.0057 (10)	0.0002 (12)	0.0160 (10)
C9	0.0395 (12)	0.0258 (9)	0.0336 (12)	0.0066 (8)	0.0006 (9)	0.0047 (8)
C10	0.0228 (9)	0.0240 (8)	0.0252 (10)	0.0056 (7)	0.0012 (8)	0.0043 (7)
C11	0.0331 (12)	0.0293 (10)	0.0357 (12)	0.0050 (8)	0.0050 (9)	−0.0045 (8)
C12	0.0542 (16)	0.0340 (11)	0.0369 (13)	0.0121 (10)	0.0130 (11)	−0.0027 (9)
C13	0.0475 (15)	0.0459 (13)	0.0439 (15)	0.0244 (11)	0.0223 (12)	0.0124 (11)
C14	0.0257 (12)	0.0579 (15)	0.0512 (16)	0.0097 (10)	0.0101 (11)	0.0150 (13)
C15	0.0258 (11)	0.0377 (11)	0.0362 (12)	0.0044 (9)	0.0003 (9)	0.0065 (9)
C16	0.0218 (9)	0.0185 (8)	0.0304 (10)	−0.0045 (7)	0.0002 (8)	−0.0020 (7)
C17	0.0202 (9)	0.0171 (7)	0.0249 (9)	−0.0031 (7)	0.0048 (7)	−0.0006 (7)
C18	0.0243 (10)	0.0193 (8)	0.0293 (11)	−0.0020 (7)	0.0073 (8)	0.0035 (7)
C19	0.0239 (9)	0.0159 (8)	0.0290 (10)	0.0026 (7)	0.0016 (8)	0.0015 (7)
C20	0.0253 (10)	0.0263 (9)	0.0268 (10)	0.0023 (7)	0.0020 (8)	0.0006 (8)
C21	0.0251 (11)	0.0352 (11)	0.0409 (13)	0.0045 (8)	0.0074 (9)	0.0023 (9)
C22	0.0263 (11)	0.0475 (13)	0.0462 (15)	0.0067 (9)	−0.0053 (10)	0.0096 (11)
C23	0.0346 (13)	0.0601 (15)	0.0339 (13)	0.0081 (11)	−0.0046 (10)	0.0128 (11)
C24	0.0295 (12)	0.0401 (11)	0.0302 (11)	0.0053 (9)	0.0034 (9)	0.0080 (9)
C25	0.0193 (9)	0.0213 (8)	0.0243 (10)	0.0019 (7)	0.0022 (7)	−0.0034 (7)
C26	0.0305 (11)	0.0245 (9)	0.0266 (10)	−0.0063 (8)	0.0024 (8)	−0.0025 (7)
C27	0.0300 (11)	0.0371 (11)	0.0268 (11)	−0.0089 (9)	0.0038 (9)	−0.0008 (8)
C28	0.0270 (10)	0.0357 (10)	0.0269 (11)	0.0002 (8)	0.0013 (8)	−0.0096 (8)
C29	0.0428 (13)	0.0224 (9)	0.0355 (12)	0.0005 (8)	−0.0020 (10)	−0.0074 (8)
C30	0.0424 (12)	0.0182 (9)	0.0300 (11)	0.0015 (8)	0.0000 (9)	−0.0002 (8)

### Geometric parameters (Å, °)

**Table:**

I1—Co2	2.5980 (6)	C11—C12	1.379 (3)
Co2—C17	2.0685 (18)	C11—H11	0.9300
Co2—P2	2.1597 (6)	C12—C13	1.384 (4)
Co2—P3	2.1734 (6)	C12—H12	0.9300
Co2—P1	2.2278 (6)	C13—C14	1.373 (4)
P1—C1	1.824 (2)	C13—H13	0.9300
P1—C3	1.8263 (19)	C14—C15	1.390 (3)
P1—C2	1.827 (2)	C14—H14	0.9300
P2—O1	1.6231 (13)	C15—H15	0.9300
P2—C4	1.823 (2)	C16—C17	1.519 (3)
P2—C10	1.829 (2)	C16—H16	1.00 (2)
P3—O2	1.6278 (14)	C16—H17	0.99 (2)
P3—C25	1.821 (2)	C17—C18	1.513 (3)
P3—C19	1.823 (2)	C17—H31	1.00 (2)
O1—C16	1.454 (2)	C18—H18	0.97 (3)
O2—C18	1.460 (2)	C18—H19	0.98 (2)
C1—H1A	0.9600	C19—C20	1.394 (3)
C1—H1B	0.9600	C19—C24	1.397 (3)
C1—H1C	0.9600	C20—C21	1.385 (3)
C2—H2A	0.9600	C20—H20	0.9300
C2—H2B	0.9600	C21—C22	1.378 (4)
C2—H2C	0.9600	C21—H21	0.9300
C3—H3A	0.9600	C22—C23	1.391 (4)
C3—H3B	0.9600	C22—H22	0.9300
C3—H3C	0.9600	C23—C24	1.389 (3)
C4—C5	1.386 (3)	C23—H23	0.9300
C4—C9	1.398 (3)	C24—H24	0.9300
C5—C6	1.392 (3)	C25—C26	1.389 (3)
C5—H5	0.9300	C25—C30	1.396 (3)
C6—C7	1.383 (4)	C26—C27	1.388 (3)
C6—H6	0.9300	C26—H26	0.9300
C7—C8	1.372 (4)	C27—C28	1.387 (3)
C7—H7	0.9300	C27—H27	0.9300
C8—C9	1.386 (3)	C28—C29	1.386 (3)
C8—H8	0.9300	C28—H28	0.9300
C9—H9	0.9300	C29—C30	1.380 (3)
C10—C11	1.389 (3)	C29—H29	0.9300
C10—C15	1.391 (3)	C30—H30	0.9300
			
C17—Co2—P2	76.51 (6)	C12—C11—C10	120.8 (2)
C17—Co2—P3	79.01 (5)	C12—C11—H11	119.6
P2—Co2—P3	114.06 (2)	C10—C11—H11	119.6
C17—Co2—P1	178.47 (6)	C11—C12—C13	120.0 (2)
P2—Co2—P1	102.26 (2)	C11—C12—H12	120.0
P3—Co2—P1	100.77 (2)	C13—C12—H12	120.0
C17—Co2—I1	91.36 (6)	C14—C13—C12	119.7 (2)
P2—Co2—I1	124.951 (18)	C14—C13—H13	120.2
P3—Co2—I1	115.788 (17)	C12—C13—H13	120.2
P1—Co2—I1	90.104 (16)	C13—C14—C15	120.7 (2)
C1—P1—C3	101.64 (10)	C13—C14—H14	119.7
C1—P1—C2	100.03 (11)	C15—C14—H14	119.7
C3—P1—C2	101.59 (10)	C14—C15—C10	119.9 (2)
C1—P1—Co2	119.56 (7)	C14—C15—H15	120.0
C3—P1—Co2	114.34 (7)	C10—C15—H15	120.0
C2—P1—Co2	116.88 (7)	O1—C16—C17	112.58 (14)
O1—P2—C4	99.72 (8)	O1—C16—H16	107.0 (14)
O1—P2—C10	102.47 (9)	C17—C16—H16	111.2 (14)
C4—P2—C10	103.81 (9)	O1—C16—H17	107.3 (14)
O1—P2—Co2	108.65 (5)	C17—C16—H17	110.3 (15)
C4—P2—Co2	128.62 (7)	H16—C16—H17	108.3 (19)
C10—P2—Co2	110.39 (7)	C18—C17—C16	109.96 (15)
O2—P3—C25	102.49 (8)	C18—C17—Co2	108.77 (13)
O2—P3—C19	99.99 (8)	C16—C17—Co2	113.12 (13)
C25—P3—C19	102.15 (9)	C18—C17—H31	109.3 (13)
O2—P3—Co2	106.24 (5)	C16—C17—H31	111.0 (13)
C25—P3—Co2	123.96 (6)	Co2—C17—H31	104.6 (12)
C19—P3—Co2	118.45 (6)	O2—C18—C17	110.07 (15)
C16—O1—P2	110.85 (12)	O2—C18—H18	108.7 (14)
C18—O2—P3	113.57 (11)	C17—C18—H18	111.6 (13)
P1—C1—H1A	109.5	O2—C18—H19	103.7 (15)
P1—C1—H1B	109.5	C17—C18—H19	113.0 (14)
H1A—C1—H1B	109.5	H18—C18—H19	109 (2)
P1—C1—H1C	109.5	C20—C19—C24	119.07 (19)
H1A—C1—H1C	109.5	C20—C19—P3	123.31 (16)
H1B—C1—H1C	109.5	C24—C19—P3	117.39 (16)
P1—C2—H2A	109.5	C21—C20—C19	120.5 (2)
P1—C2—H2B	109.5	C21—C20—H20	119.7
H2A—C2—H2B	109.5	C19—C20—H20	119.7
P1—C2—H2C	109.5	C22—C21—C20	120.1 (2)
H2A—C2—H2C	109.5	C22—C21—H21	120.0
H2B—C2—H2C	109.5	C20—C21—H21	120.0
P1—C3—H3A	109.5	C21—C22—C23	120.2 (2)
P1—C3—H3B	109.5	C21—C22—H22	119.9
H3A—C3—H3B	109.5	C23—C22—H22	119.9
P1—C3—H3C	109.5	C24—C23—C22	119.9 (2)
H3A—C3—H3C	109.5	C24—C23—H23	120.1
H3B—C3—H3C	109.5	C22—C23—H23	120.1
C5—C4—C9	119.02 (19)	C23—C24—C19	120.2 (2)
C5—C4—P2	120.07 (15)	C23—C24—H24	119.9
C9—C4—P2	120.74 (17)	C19—C24—H24	119.9
C4—C5—C6	120.1 (2)	C26—C25—C30	118.65 (19)
C4—C5—H5	119.9	C26—C25—P3	120.75 (14)
C6—C5—H5	119.9	C30—C25—P3	120.55 (16)
C7—C6—C5	120.4 (2)	C27—C26—C25	120.79 (18)
C7—C6—H6	119.8	C27—C26—H26	119.6
C5—C6—H6	119.8	C25—C26—H26	119.6
C8—C7—C6	119.5 (2)	C28—C27—C26	119.9 (2)
C8—C7—H7	120.3	C28—C27—H27	120.1
C6—C7—H7	120.3	C26—C27—H27	120.1
C7—C8—C9	120.8 (2)	C29—C28—C27	119.6 (2)
C7—C8—H8	119.6	C29—C28—H28	120.2
C9—C8—H8	119.6	C27—C28—H28	120.2
C8—C9—C4	120.0 (2)	C30—C29—C28	120.38 (19)
C8—C9—H9	120.0	C30—C29—H29	119.8
C4—C9—H9	120.0	C28—C29—H29	119.8
C11—C10—C15	118.9 (2)	C29—C30—C25	120.6 (2)
C11—C10—P2	121.34 (16)	C29—C30—H30	119.7
C15—C10—P2	119.59 (16)	C25—C30—H30	119.7
			
P2—Co2—P1—C1	−60.80 (9)	C4—P2—C10—C11	−89.34 (18)
P3—Co2—P1—C1	56.98 (9)	Co2—P2—C10—C11	51.66 (18)
I1—Co2—P1—C1	173.28 (9)	O1—P2—C10—C15	−7.46 (18)
P2—Co2—P1—C3	59.95 (8)	C4—P2—C10—C15	95.97 (17)
P3—Co2—P1—C3	177.72 (8)	Co2—P2—C10—C15	−123.03 (16)
I1—Co2—P1—C3	−65.97 (8)	C15—C10—C11—C12	−1.5 (3)
P2—Co2—P1—C2	178.41 (8)	P2—C10—C11—C12	−176.23 (18)
P3—Co2—P1—C2	−63.81 (9)	C10—C11—C12—C13	1.1 (4)
I1—Co2—P1—C2	52.49 (8)	C11—C12—C13—C14	0.0 (4)
C17—Co2—P2—O1	−34.20 (8)	C12—C13—C14—C15	−0.8 (4)
P3—Co2—P2—O1	37.03 (6)	C13—C14—C15—C10	0.4 (4)
P1—Co2—P2—O1	144.87 (6)	C11—C10—C15—C14	0.7 (3)
I1—Co2—P2—O1	−116.27 (6)	P2—C10—C15—C14	175.55 (18)
C17—Co2—P2—C4	−154.04 (10)	P2—O1—C16—C17	3.5 (2)
P3—Co2—P2—C4	−82.82 (9)	O1—C16—C17—C18	−155.92 (16)
P1—Co2—P2—C4	25.02 (9)	O1—C16—C17—Co2	−34.09 (19)
I1—Co2—P2—C4	123.89 (8)	P2—Co2—C17—C18	160.80 (14)
C17—Co2—P2—C10	77.43 (9)	P3—Co2—C17—C18	42.53 (12)
P3—Co2—P2—C10	148.66 (7)	I1—Co2—C17—C18	−73.49 (13)
P1—Co2—P2—C10	−103.50 (7)	P2—Co2—C17—C16	38.31 (12)
I1—Co2—P2—C10	−4.64 (7)	P3—Co2—C17—C16	−79.97 (13)
C17—Co2—P3—O2	−27.71 (8)	I1—Co2—C17—C16	164.01 (12)
P2—Co2—P3—O2	−97.41 (6)	P3—O2—C18—C17	23.9 (2)
P1—Co2—P3—O2	153.84 (6)	C16—C17—C18—O2	75.7 (2)
I1—Co2—P3—O2	58.45 (6)	Co2—C17—C18—O2	−48.69 (18)
C17—Co2—P3—C25	90.18 (10)	O2—P3—C19—C20	135.14 (16)
P2—Co2—P3—C25	20.48 (8)	C25—P3—C19—C20	29.91 (18)
P1—Co2—P3—C25	−88.28 (8)	Co2—P3—C19—C20	−110.13 (16)
I1—Co2—P3—C25	176.33 (7)	O2—P3—C19—C24	−50.42 (17)
C17—Co2—P3—C19	−139.02 (9)	C25—P3—C19—C24	−155.65 (16)
P2—Co2—P3—C19	151.28 (7)	Co2—P3—C19—C24	64.31 (17)
P1—Co2—P3—C19	42.53 (8)	C24—C19—C20—C21	−0.1 (3)
I1—Co2—P3—C19	−52.86 (8)	P3—C19—C20—C21	174.23 (15)
C4—P2—O1—C16	162.61 (13)	C19—C20—C21—C22	−0.1 (3)
C10—P2—O1—C16	−90.78 (14)	C20—C21—C22—C23	−0.1 (4)
Co2—P2—O1—C16	26.04 (13)	C21—C22—C23—C24	0.5 (4)
C25—P3—O2—C18	−122.07 (14)	C22—C23—C24—C19	−0.7 (4)
C19—P3—O2—C18	132.99 (14)	C20—C19—C24—C23	0.5 (3)
Co2—P3—O2—C18	9.27 (15)	P3—C19—C24—C23	−174.15 (18)
O1—P2—C4—C5	−35.28 (18)	O2—P3—C25—C26	166.52 (16)
C10—P2—C4—C5	−140.81 (17)	C19—P3—C25—C26	−90.21 (17)
Co2—P2—C4—C5	88.23 (17)	Co2—P3—C25—C26	46.88 (19)
O1—P2—C4—C9	149.46 (17)	O2—P3—C25—C30	−15.99 (19)
C10—P2—C4—C9	43.93 (19)	C19—P3—C25—C30	87.28 (18)
Co2—P2—C4—C9	−87.03 (18)	Co2—P3—C25—C30	−135.62 (15)
C9—C4—C5—C6	0.6 (3)	C30—C25—C26—C27	−3.5 (3)
P2—C4—C5—C6	−174.71 (17)	P3—C25—C26—C27	174.08 (17)
C4—C5—C6—C7	0.9 (3)	C25—C26—C27—C28	1.8 (3)
C5—C6—C7—C8	−0.8 (4)	C26—C27—C28—C29	1.2 (3)
C6—C7—C8—C9	−0.7 (4)	C27—C28—C29—C30	−2.6 (3)
C7—C8—C9—C4	2.2 (4)	C28—C29—C30—C25	0.9 (4)
C5—C4—C9—C8	−2.2 (3)	C26—C25—C30—C29	2.1 (3)
P2—C4—C9—C8	173.15 (18)	P3—C25—C30—C29	−175.47 (18)
O1—P2—C10—C11	167.23 (17)		
